# Supplementary material for: Safety and Immunogenicity of the Nonavalent Human Papillomavirus Vaccine in Women Living with HIV
Source: Vaccines (Basel). 2024 Jul 25;12(8):838. doi: 10.3390/vaccines12080838 (PMC11359547; doi:10.3390/vaccines12080838)
Supplement: Supplementary file 1 [file vaccines-12-00838-s001.zip › vaccines-3069228-supplementary.pdf]

vaccines

MDPI

Table S1. HPV genotypes isolated in anal mucosa at baseline that are not included in the Gardasil-9 vaccine.

Variables	n = 122
Other HPV genotypes	
HPV 32	1 (0.8)
HPV 35	6 (4.9)
HPV 39	3 (2.5)
HPV 40	1 (0.8)
HPV 42	7 (5.7)
HPV 43	2 (1.6)
HPV 44/55	8 (6.5)
HPV 51	10 (8.2)
HPV 53	3 (2.5)
HPV 54	1 (0.8)
HPV 56	5 (4.1)
HPV 59	3 (2.5)
HPV 61	6 (4.9)
HPV 62	1 (0.8)
HPV 62/81	9 (7.4)
HPV 66	5 (4.1)
HPV 67	2 (1.6)
HPV 68	9 (7.4)
HPV 70	5 (4.1)
HPV 71	2 (1.6)
HPV 72	1 (0.8)
HPV 73	4 (3.3)
HPV 81	1 (0.8)
HPV 82	1 (0.8)
HPV 84	1 (0.8)

Table S2. HPV genotypes isolated in cervical mucosa at baseline that are not included in the Gardasil-9 vaccine

Variables	n = 122
Other HPV genotypes	
HPV 19	1(0.8)
HPV 35	1 (0.8)
HPV 39	2 (1.6)
HPV 40	2 (1.6)
HPV 42	8 (6.6)
HPV 43	1 (0.8)
HPV 44	6 (4.9)
HPV 51	3 (2.5)
HPV 53	1 (0.8)
HPV 56	2 (1.6)
HPV 61	4 (3.3)
HPV 62	2 (1.6)
HPV 62/81	9 (7.4)
HPV 64	1 (0.8)
HPV 66	3 (2.5)
HPV 68	3 (2.5)
HPV 70	2 (1.6)
HPV 72	4 (3.3)
HPV 73	6 (4.9)
HPV 81	2 (1.6)
HPV 84	1 (0.8)

Table S3. Univariate and multivariable analysis of factors associated with anal HR-HPV infection.

	WLHIV without HR-HPV N=73	WLHIV with HR-HPV N= 48	P*	HR; (95% CI)
Age; mean years (±SD)	50.5 (7.6)	48.2 (11.8)	0.19	1.02 (0.93-1.11)
Educational level, n (%)				1.71 (0.27-10.58)
Illiterate	11 (15.1)	9 (19.1)	0.79	
Elementary	35 (47.9)	21 (44.7)		
High school	22 (30.1)	12 (25.5)		
University education	5 (6.8)	5 (10.6)		
Sex in the previous 12 months, n (%)	52 (71.2)	31 (64.6)	0.44	1.09 (0.19-6.15)
Anal sex previous 12 months, n (%)	12 (16.4)	9 (18.8)	0.74	
Sex worker	12 (16.4)	8 (16.7)	0.97	
Partners in the previous 12 months; median (IQR)	1 (0-1)	1 (0-1)	0.96	
Life-time partners; median (IQR)	3 (2-9)	6 (4-14)	0.04	1 (0.999-1.001)
Time since commencement of sexual activity (years); median (IQR)	33 (27-37)	30 (18-41)	0.96	
Condom use, n (%)	33 (45.2)	19 (39.6)	0.86	1.21(0.28-5.2)
Rate of use condom; median (IQR)	0 (0-100)	0 (0-100)	0.87	
Retired	16 (21.9)	13 (28.3)	0.38	
Polypharmacy	14 (19.2)	11 (22.9)	0.62	
Perianal/genital warts, n (%)	1 (1.4)	4 (8.7)	0.71	
History of anal/genital warts, n (%)	9 (12.3)	4 (8.5)	0,55	
History of syphilis, n (%)	4 (5.5)	2 (4.2)	1	
History others STD	8 (11.3)	4 (8.7)	0.76	1.88 (0.35-10)
CMV IgG positive, n (%)	53 (72.6)	40 (87)	0.06	
Smoking, n (%)	38 (52.1)	27(56.3)	0.65	1.25 (0.28-5.5)
Smoking, packets/year; median (IQR)	9(0-26)	2.7 (0-14)	0.2	
Ex-UDI, n (%)	16 (21.9)	11 (22.9)	0.89	
Duration of HIV (year); median (IQR)	22.7 (14.3-27)	12.2(5.4-20.1)	0.03	0.99 (0.98-1.008)
CD4 mean nadir (cells/μL); mean (± SD)	226.4 (178)	221.1 (178.3)	0.9	1.002 (0.996-1.008)
Treatment naïve, n (%)	0	1(2.1)	0.39	
VL of HIV, log10; mean (± SD)	1.85 (2.7)	3.8 (4.6)	0.33	
VL < 50 copies/mL, n (%)	70 (94.6)	42 (87.5)	0.153	3.22 (0.25-40.84)
Virological failure	0	1 (2.1)	0.39	
CD4 mean; cells/μL, mean (± SD)	854.6 (351.2)	725.1 (369.4)	0.054	0.99 (0.99-1.001)
CD8 mean; cells/μL, mean (± SD)	755.4 (310.4)	835.8 (625.1)	0.412	
CD4/CD8 ratio	1.3 (0.6)	1.1 (0.77)	0.25	
History AIDS-defining illnesses; n (%)	36 (50)	22 (46.8)	0.73	0.81 (0.14-4.76)

Median duration of ART (year), (IQR)	16.3 (12.3-24)	11.5 (4.3-19.5)	0.54	0.995 (0.985-1.006)
Triple therapy, n (%)	32 (43.8)	21 (43.8)	0.99	
Dual therapy, n (%)	35 (47.9)	22 (45.8)	0.82	
Monotherapy (DRV/cob), n (%)	6 (8.2)	4 (8.3)	1	
Number of TAR, median (IQR)	4(4-6)	4 (2-5.5)	0.06	
Median duration of current ART (months), (IQR)	21.5 (7.5-34.5)	16 (6-35.5)	0.537	0.92 (0.64-1.3)
BIC/FTC/TAF	25 (34.2)	9 (18.8)	0.06	
DRV/cob/FTC/TAF	4 (5.5)	6 (12.6)	0.19	0.26(0.05-1.2)
DTG/ABC /3TC	1 (1.4)	2 (4.2)	0.56	
DTG/3TC	11 (15.1)	10 (21.8)	0.41	
DTG/RPV	12 (16.4)	6 (12.5)	0.55	
DRV/cob	15 (20.5)	12 (25)	0.56	
Active chronic HCV infection, n (%)	1 (1.4)	0	1	
Cured HCV infection, n (%)	19 (26)	12 (25)	1	
Active chronic HBV infection (positive surface Ag), n (%)	2 (2.7)	0	0.52	
History of cervical dysplasia, n (%)	16 (22.9)	11 (24.4)	0.85	1.93 (0.47-7.98)
-CIN 1	6	3	1	
-CIN 2	4 (5.5)	4 (8.9)	0.7	
-CIN 3	5 (7.1)	4 (8.9)	0.74	
-Cancer	1 (1.4)	1 (2.2)	1	
History of vulvar dysplasia, n (%)	1 (1.4)	1 (2.2)	1	
History of cervical HR-HPV infection, n (%)	3 (4.8)	5 (12.5)	0.26	
Anal LR-HPV, n (%)	13 (18.1)	30 (62.5)	0.0001	2.6 (0.46-14.5)
Median of anal LR-HPV genotypes, IQR	0 (0-0)	1 (0-1)	0.0001	
Gynaecologic Infection by HR-HPV, n (%)	10 (13.7)	21 (43.8)	0.0001	5.027 (1.009-25.042)
Median of HR-HPV genotypes, IQR	0 (0-0)	1(0-1)	0.0001	
Gynaecologic infection by LR-HPV, n (%)	12 (16.4)	19 (39.6)	0.004	1.8(0.22-15.3)
Median of LR-HPV genotypes, IQR	0 (0-0)	0 (0-1)	0.02	0.71(0.02-20.26)
Gynaecologic due to HR and LR-HPV	4 (5.5)	8 (16.7)	0.06	
Gynaecologic dysplasia, n (%)				
-CIN 1	0	2 (8)	0.15	
-CIN 2	1 (2.6)	1 (4)	1	
Antibodies against HPV positive, n (%)	7 (9.6)	3 (6.3)	0.704	

P*: p-value; $p < 0.05$; Ex-UDI: ex-user drug injection; WLHIV: women living with HIV; BICT/ FTC /TAF/. bictegravir/ emtricitabine/tenofovir alafenamide; DRV/cob/FTC/TAF: darunavir/cobicistat/ tenofovir alafenamide; DTG/ ABC/ 3TC: dolutegravir/abacavir/lamivudine; DTG/3TC: dolutegravir/lamivudine; DTG/RPV: dolutegravir/rilpivirine; DRV/cob: darunavir/cobicistat.
